# Persistent Low Level of Osterix Accelerates Interleukin-6 Production and Impairs Regeneration after Tissue Injury

**DOI:** 10.1371/journal.pone.0069859

**Published:** 2013-07-26

**Authors:** Wook-Young Baek, Seung-Yoon Park, Yeo Hyang Kim, Min-A Lee, Tae-Hwan Kwon, Kwon-Moo Park, Benoit de Crombrugghe, Jung-Eun Kim

**Affiliations:** 1 Cell and Matrix Research Institute, Kyungpook National University, Daegu, Korea; 2 Department of Molecular Medicine, Kyungpook National University School of Medicine, Daegu, Korea; 3 Department of Biochemistry and Cell Biology, Kyungpook National University School of Medicine, Daegu, Korea; 4 Department of Anatomy, Kyungpook National University School of Medicine, Daegu, Korea; 5 Department of Biochemistry, School of Medicine, Dongguk University, Gyeongju, Korea; 6 Department of Pediatrics, Keimyung University School of Medicine, Daegu, Korea; 7 Department of Genetics, University of Texas M. D. Anderson Cancer Center, Houston, Texas, United States of America; INSERM, UMR-S747, France

## Abstract

Osterix (Osx) is an essential transcription factor for osteoblast differentiation and bone formation. Osx knockout show a complete absence of bone formation, whereas Osx conditional knockout in osteoblasts produce an osteopenic phenotype after birth. Here, we questioned whether Osx has a potential role in regulating physiological homeostasis. In Osx heterozygotes expressing low levels of Osx in bones, the expression levels of pro-inflammatory cytokines were significantly elevated, indicating that reduced Osx expression may reflect an inflammatory-prone state. In particular, the expression of interleukin-6, a key mediator of chronic inflammation, was increased in Osx heterozygotes and decreased in Osx overexpressing osteoblasts, and transcriptionally down-regulated by Osx. Although no significant differences were revealed in renal morphology and function between Osx heterozygotes and wild-type under normoxic conditions, recovery of kidneys after ischemic damage was remarkably delayed in Osx heterozygotes, as indicated by elevated blood urea nitrogen and creatinine levels, and by morphological alterations consistent with acute tubular necrosis. Eventually, protracted low Osx expression level caused an inflammatory-prone state in the body, resulting in the enhanced susceptibility to renal injury and the delayed renal repair after ischemia/reperfusion. This study suggests that the maintenance of Osx expression in bone is important in terms of preventing the onset of an inflammatory-prone state.

## Introduction

Inflammation, which is classified as either acute or chronic, is part of the body’s defense mechanism that protects against damaging stimuli or infections and plays a central role in many diseases. Whereas acute inflammation refers to the initial protective response to tissue injury, chronic inflammation refers to an imbalanced inflammatory response to tissue damage caused by persistent infections, prolonged exposure to potentially toxic agents, or autoimmunity. Chronic inflammation is of longer duration and is histologically characterized by fibrosis and angiogenesis. Several studies have demonstrated that chronic inflammation is closely linked to disease susceptibility. For example, in an allergic asthma animal model, the risk of acute myocardial ischemia/reperfusion (I/R) injury is significantly enhanced by this condition [1]. Chronic inflammation systemically upregulates pro-inflammatory cytokines and exacerbates ischemic brain injuries [2]. Furthermore, a chronic inflammatory mouse model with sickle cell disease is highly sensitive to renal I/R injury [3]. Chronic inflammation also delays wound healing and increases scarring [4]. These observations indicate that the inflammatory-prone state can worsen the damage from I/R injury and can delay wound repair in a wide range of tissues.

Cytokines are produced and secreted by osteoblasts and various immune cells, and participate in the regulation of immune responses to diseases and infections that lead to inflammation [5], [6], [7], [8]. Pro-inflammatory cytokines are associated with the development of various diseases, such as, rheumatoid arthritis, diabetes, and cancer [9]. In particular, the pro-inflammatory cytokine interleukin (IL)-6 plays multiple roles during infections and injuries, and serves as a reliable clinical indicator of the risks for various diseases [10]. For example, serum IL-6 levels are increased in patients with cardiovascular problems and in Alzheimer’s disease, rheumatoid arthritis, and chronic kidney disease (CKD), thereby suggesting that elevated levels of IL-6 contribute to chronic inflammation [11], [12], [13], [14], [15], [16]. Chronic inflammation induced by IL-6 and IL-1β accelerates the degradation of insulin-like growth factor I binding protein 3 (IGFBP-3), resulting in growth retardation [17]. Macrophage inflammatory protein 1 alpha (MIP-1α) participates in pro-inflammatory activities by recruiting inflammatory cells and inducing the productions of IL-6 and TNF-α [18], [19]. Furthermore, the increased expression of MIP-1α has been linked to the progression of inflammatory diseases [20]. Recently, Cao et al. [21] reported that bone transcription factor Osterix (Osx) represses IL-1α transcription and downregulates IL-1α expression in K7M2 mouse osteosarcoma cells. However, the regulatory associations between bone transcription factors and pro-inflammatory cytokines that lead to chronic inflammation have not been sufficiently determined. Herein, we questioned whether Osx modulates the production and secretion of pro-inflammatory cytokines. In Osx heterozygotes expressing Osx at lower levels than wild-type controls, cortical thicknesses (as determined by quantitative computed tomography (QCT)) and the in vitro differentiation of primary calvarial osteoblasts were remarkably reduced. Furthermore, in these mice, the expression levels of pro-inflammatory cytokines were significantly increased. In particular, the expression of IL-6 was increased in bones of Osx heterozygotes and decreased in Osx overexpressing osteoblasts, indicating the presence of an inflammatory-prone state due to diminished Osx expression. In this study, we investigated the relationship between Osx gene dosage and IL-6 levels with respect to healing in a renal I/R injury model. The transcriptional activity of the IL-6 promoter was remarkably reduced in the presence of Osx. In Osx heterozygous mice with a normal renal morphology and function, the kidney demonstrated delayed repair and morphologic alterations consistent with acute tubular necrosis after I/R injury. These results indicated that the bone-specific transcription factor Osx is a key negative regulator of IL-6 expression in osteoblasts, and that the upregulation of IL-6 in Osx heterozygotes delays the healing process of the kidney after renal I/R injury. These observations suggest a novel connection between bone formation and inflammation response via an Osx-IL-6 relationship. Finally, this study suggests that the maintenance of Osx expression levels in bone importantly prevents the establishment of an inflammatory-prone state and regulates physiological homeostasis.

## Materials and Methods

### Animals

All animal procedures were reviewed and approved by the animal ethics committee of Kyungpook National University (Approval No. KNU-2010-91). They were bred and maintained under pathogen-free conditions according to the guidelines of animal facility issued by Kyungpook National University School of Medicine. Wild-type (Osx^flox/+^) and Osx heterozygotes (Osx^flox/−^) were used for experiments [22], [23]. PCR genotyping was conducted using tail genomic DNA obtained at 10 days after birth. The primer sets and PCR amplification conditions have been described previously [22].

### Calvarial Cell Culture

Primary osteoblasts were isolated from calvaria of neonatal mice and digested with 0.1% collagenase at 37°C for 30 min. Cells were plated onto 24-well culture dishes at a density of 1×10^5^ cells/well and differentiated in vitro in conditioned medium supplemented with 10% fetal bovine serum (FBS) plus 100 µg/ml ascorbic acid and 5 mM β-glycerophosphate. After 3 wk of culture, mineralized bone nodules were identified by alizarin red S and von Kossa staining.

### Analysis of mRNA Expression

Total RNA was isolated from long bones, kidneys, and cultured cells using Tri reagent (Invitrogen, Camarillo, CA, USA) and 2 µg aliquots were used to synthesize cDNA using Reverse Transcriptase Premix (Elpis-Biotech, Daejeon, Korea) according to the manufacturer’s instructions. For quantitative real-time PCR (qRT-PCR), the 2X SYBR Green Master Mix (Applied Biosystems) was used. The primers used for real-time PCR were as follows: Osx, 5′-CGT CCT CTC TGC TTG AGG AA-3′ and 5′-CTT GAG AAG GGA GCT GGG TA-3′; IL-1α, 5′-AAG TTT GTC ATG AAT GAT TCC CTC-3′ and 5′-GTC TCA CTA CCT GTG ATG AGT-3′; IL-6, 5′-TGT ATG AAC AAC GAT GAT GCA CTT-3′ and 5′-ACT CTG GCT TTG TCT TTC TTG TTA TC-3′; MIP-1α, 5′-GCC CTT GCT GTT CTT CTC TGT-3′ and 5′-GGC ATT CAG TTC CAG GTC AGT-3′; GM-CSF, 5′-TGT GGC TGC AGA ATT TAC-3′ and 5′-GCT GTC TAT GAA ATC CGC-3′; RANTES, 5′-CCT CAC CAT CAT CCT CAC-3′ and 5′-GCT CAT CTC CAA ATA GTT G-3′; TNF-α, 5′-TCC AGG TCT TTC AGG A-3′ and 5′-GGT AGG GCA GTA TCG -3′; and interferon-γ (IFN-γ), 5′-GAT ATC TCG AGG AAC TGG CAA AA-3′ and 5′-CTT CAA AGA GTC TGA GGT AGA AAG AGA TAA T-3′. The gene expression levels were standardized by parallel qRT-PCR using primers for glyceraldehyde 3-phosphate dehydrogenase (GAPDH). To determine the mRNA expressions of genes, RT-PCR was performed using the following primers: Osx, 5′-CGT CCT CTC TGC TTG AGG AA-3′ and 5′-CTT GAG AAG GGA GCT GGG TA-3′; IL-6, 5′-ATG AAG TTC CTC TCT GCA AGA GAC T-3′ and 5′-CAC TAG GTT TGC CGA GTA GAT CTC-3′; B-cell lymphoma 2 (Bcl-2), 5′-AGA GGG GCT ACG AGT GGG AT-3′ and 5′-CTC AGT CAT CCA CAG GGC GA-3′; nuclear factor kappa B (NF-κB), 5′-AGG AAG AAA ATG GCG GAG TT-3′ and 5′-GCA TAA GCT TCT GGC GTT TC-3′; monocyte chemotactic protein (MCP), 5′-ATG CAG GTC CCT GTC ATG-3′ and 5′-GCT TGA GGT GGT TGT GGA-3′; tumor necrosis factor alpha (TNF-α), 5′-AGA GGC TGG AGA TGC AGA ACG-3′ and 5′-AAG GAA GTG GCT ACC AGC TCG-3′; connective tissue growth factor (CTGF), 5′-CTG GAC GGC TGC GGC TGC TG-3′ and 5′-GGT CCT TGG GCT CGT CAC AC-3′; Heme oxygenase 1 (HO-1), 5′-GAA GGA GGC CAC CAA GGA GG-3′ and 5′-GTG CTG TGT GGC TGG CGT GC-3′; IL-1α, 5′-AAG TTT GTC ATG AAT GAT TCC CTC-3′ and 5′-GTC TCA CTA CCT GTG ATG AGT-3′; IL-1β, 5′-TTG ACG GAC CCC AAA AGA TG-3′ and 5′-AGA AGG TGC TCA TGT CCT CA-3′. Gene expressions were normalized versus GAPDH. Image J software (NIH, Bethesda, MD) was used to quantify the expression levels of RT-PCR.

### Mouse Cytokine/Chemokine Assay

To conduct the serum cytokine/chemokine assays, blood samples were obtained by cardiac puncture from mice at 7 wk of age. To collect serum, blood was incubated at room temperature for 5 min and centrifuged at 6,000 rpm for 5 min. The concentrations of IL-1α and IL-6 were measured using the Milliplex map mouse cytokine/chemokine kit (Millipore Corp., St. Charles, USA).

### Transient Transfection and Luciferase Reporter Assay

Mouse osteoblast-like MC3T3-E1 cells were cultured in α-modified essential medium (α-MEM) supplemented with 10% FBS and 100 U/mL of penicillin at 37°C in a humidified atmosphere containing 5% CO2. To overexpress Osx, an Osx cDNA construct was transfected into cells at a density of 2.5×10^5^ cells/ml using Lipofectamine™ 2000 (Invitrogen, Camarillo, CA, USA) according to the manufacturer’s protocol. After 5 h of transfection, the cells were placed in 10% FBS containing medium and incubated until harvest. To assay the transcriptional activity of IL-6, the 1300 bp promoter upstream of mouse IL-6 was used. This construct was generously provided by Dr. Gail A. Bishop [24]. MC3T3-E1 cells were transfected with empty vector, full-length (1300 bp) IL-6 promoter construct, or truncated IL-6 promoter constructs, IL-6 luciferase reporter −231 and −84 bp. The truncated constructs were also donated by Dr. Gail A. Bishop [24]. After growing the cells in 12-well plates at a density of 8×10^4^ cells/well, they were transiently transfected with 300 ng of IL-6 promoter using Lipofectamine™ 2000 (Invitrogen). Co-transfection was performed by addition of the Osx-expressing construct or the empty vector. After 5 h of transfection, the cells were recovered with 10% FBS containing medium and incubated in this medium until harvest. The cells were incubated in the presence of absence of lipopolysaccharide (LPS, Sigma) for 6 h before harvest. Luciferase activity was measured using the dual-luciferase reporter assay system (Promega, Madison, WI), according to the manufacturer’s instructions.

### Chromatin Immunoprecipitation (ChIP) Assay

ChIP was performed using an UPSTATE Kit (Millipore, Billerica, MA, USA), according to the manufacturer’s instructions. Briefly, osteoblastic MC3T3-E1 cells were seeded at a density of 1×10^6^ cells on a 10 cm dish and transfected with Osx cDNA for 6 h. After transfection, the cells were fixed with 1% formaldehyde, washed with PBS, and resuspended in SDS lysis buffer. The cells were then sonicated for 28 cycles of 15 sec pulse and 60 sec recovery to yield DNA fragments ranging from 150 bp to 1 kb. After centrifuging the sonicated samples, the supernatant was diluted 5-fold into ChIP dilution buffer, and then precleared by incubating with salmon sperm DNA/protein G-agarose-50% slurry for 2 h at 4°C. The contents of the supernatant were then immunoprecipitated with 1 µg of anti-Osx antibody (Abcam) or isotype-matched control antibody (anti-Osteocrin, Abcam) overnight at 4°C. Immunoprecipitated complexes were eluted from protein G-agarose beads by incubation at room temperature for 10 min with gentle agitation in elution buffer (1% SDS, 0.1 M NaHCO_3_). The genomic DNAs that had separated from the proteins were then used as templates for PCR using the following specific primers: IL-6 forward primer, 5′-GAC TTG GAA GCC AAG ATT GC-3′ and IL-6 reverse primer, 5′-ACC CAA CCT GGA CAA CAG AC-3′. As negative controls, GAPDH primers were used as follows: GAPDH forward primer, 5′-TGC CAC CCA GAA GAC TGT G-3′ and GAPDH reverse primer, 5′-ATG TAG GCC ATG AGG TCC AC-3′.

### Histological Examination of Kidneys and Clearance Studies for Renal Function

After perfusion with sterile PBS, kidneys were fixed in 4% paraformaldehyde, embedded in paraffin, and sectioned at 2 µm. The sections were stained with toluidine blue for 5 min to evaluate kidney morphologies. Serum and urine samples were collected from mice for clearance studies examining renal function. Mice were kept in metabolic cages to allow quantitative urine collection. Serum was collected by cardiac puncture at the time of sacrifice. Serum and urine osmolality, creatinine levels, and blood urea nitrogen (BUN) levels were measured. The serum concentrations of sodium and potassium, and the serum and urine concentration of calcium and phosphate were also measured.

### Induction of Kidney Ischemia/Reperfusion (I/R) Injury

Mice were intraperitoneally anesthetized with pentobarbital sodium at 60 mg/kg body weight at 7 wk of age. Both renal pedicles were clamped for 30 min using nontraumatic microaneurysm clamps through flank incisions to induce ischemia (Roboz). Renal reperfusion was visually confirmed after removal of the clamps. The kidneys were then harvested at the times indicated in the figures for biochemical studies or histological analysis. To assess kidney function, blood was taken from the retrobulbar vein plexus at the times indicated in figures. The concentrations of BUN and plasma creatinine were measured using a BUN assay kit (Asan PHARM Co. LTD, Gyeonggi-Do, Korea) and a Beckman Creatinine Analyzer II (Beckman), respectively.

### Statistical Analysis

Statistical differences between the groups were analyzed with the unpaired *t*-test and *p* values less than 0.05 were considered significant.

## Results

### Inflammatory-prone Condition in Osx Heterozygotes

**Figure 1:**
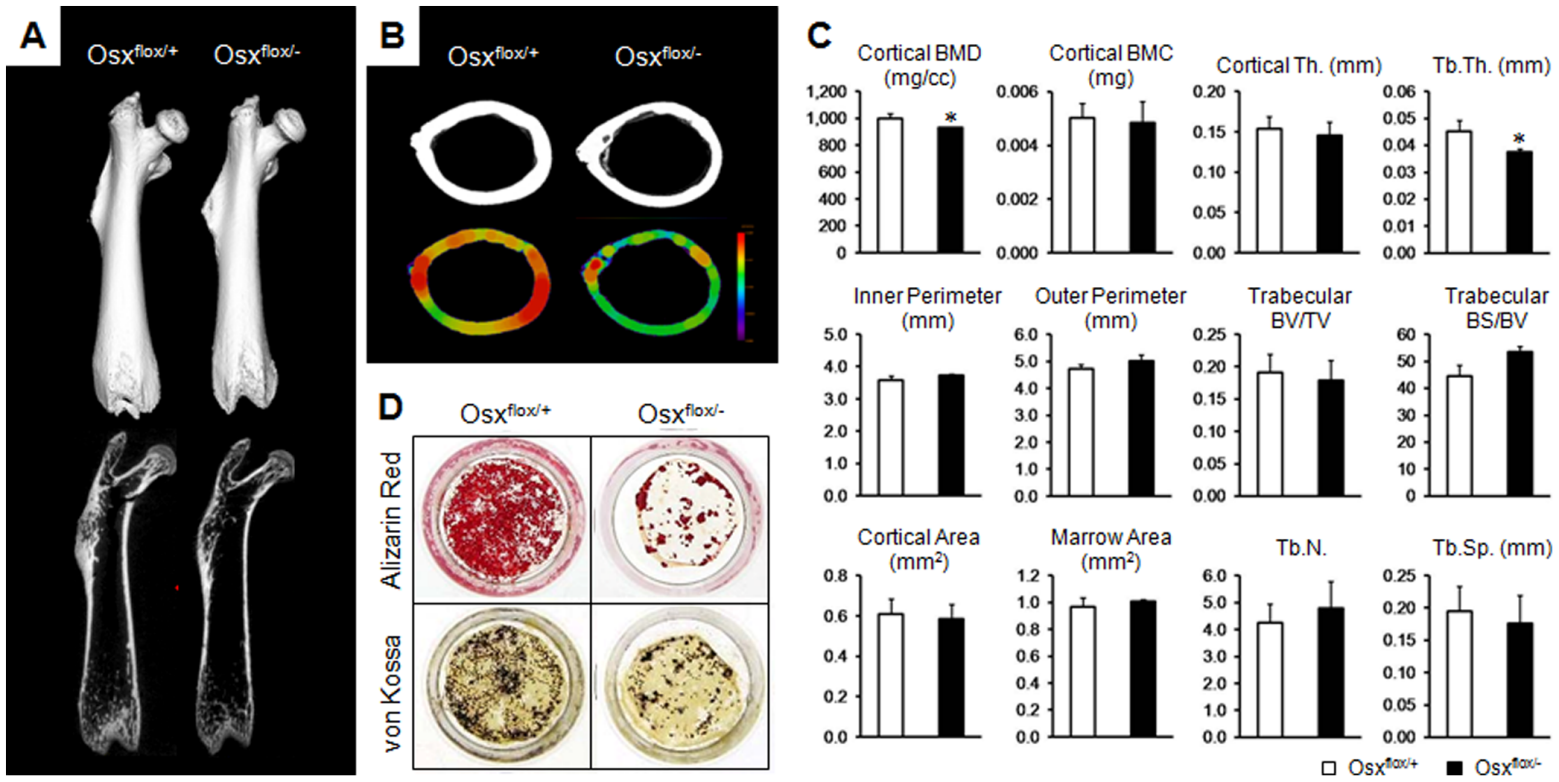
Reduced osteoblast function in bone formation in Osx heterozygotes. (A) Qualitative 3D and 2D µCT images of femoral bone at 8 wk of age. No differences in femoral length and morphology were observed between wild-type (Osx^flox/+^) and Osx heterozygous (Osx^flox/−^) mice. (B) µCT analysis in diaphysial transverse sections of femoral bone. Colored regions indicate the cortical thickness of femoral diaphyses as measured by peripheral QCT. The thickness of cortical bone was significantly reduced in Osx heterozygotes. (C) Histomorphometrical analysis of µCT images. Compared with wild-type, Osx heterozygous mice showed a significant reduction of cortical bone mineral density (BMD) and trabecular thickness (Tb.Th). BMC, bone mineral contents; BV/TV, bone volume per tissue volume; BS/BV, bone surface to bone volume; Tb.N, trabecular number; Tb.Sp, trabecular separation. *p<0.05 versus wild-type. (D) In vitro osteoblastic differentiation and mineralization in primary calvarial osteoblasts. Osteoblastic differentiation and deposited calcium were visualized by alizarin red S and von Kossa staining. Mineralized bone nodules were significantly decreased in Osx heterozygous mice.

To investigate whether osteoblasts expressing low levels of Osx function normally in matrix mineralization and bone formation, in vivo and in vitro bone formation were examined in wild-type (Osx^flox/+^) and Osx heterozygous (Osx^flox/−^) mice. Even though no differences in femoral lengths and phenotypes were observed by quantitative 3D and 2D µCT analyses between the two populations of mice at 8 wk of age (Fig. 1A), a reduction in the cortical bone thickness was observed in the femoral diaphyses of Osx heterozygotes by peripheral QCT (Fig. 1B). Bone morphometric parameters were calculated at cortical and trabecular regions using the eXplore MicroView version 2.2 (GE Healthcare). Cortical bone mineral density (BMD) and trabecular thickness (Tb.Th) were significantly decreased in Osx heterozygotes compared to wild-type, while other cortical or trabecular parameters were not considerably different between two groups (Fig. 1C). An in vitro assay of differentiation with primary calvarial osteoblasts in Osx heterozygotes revealed a dramatic reduction in mineralized nodule formation in this population, as shown by alizarin red and von Kossa staining (Fig. 1D). Expressions of osteoblast markers were obviously reduced in Osx heterozygotes, indicating that osteoblasts with low Osx expression have a reduced function for bone formation and mineralization (Fig. S1A, B). These results indicated that the reduced osteoblast function for bone formation was due to the low level of Osx expression in bones of Osx heterozygotes.

**Figure 2:**
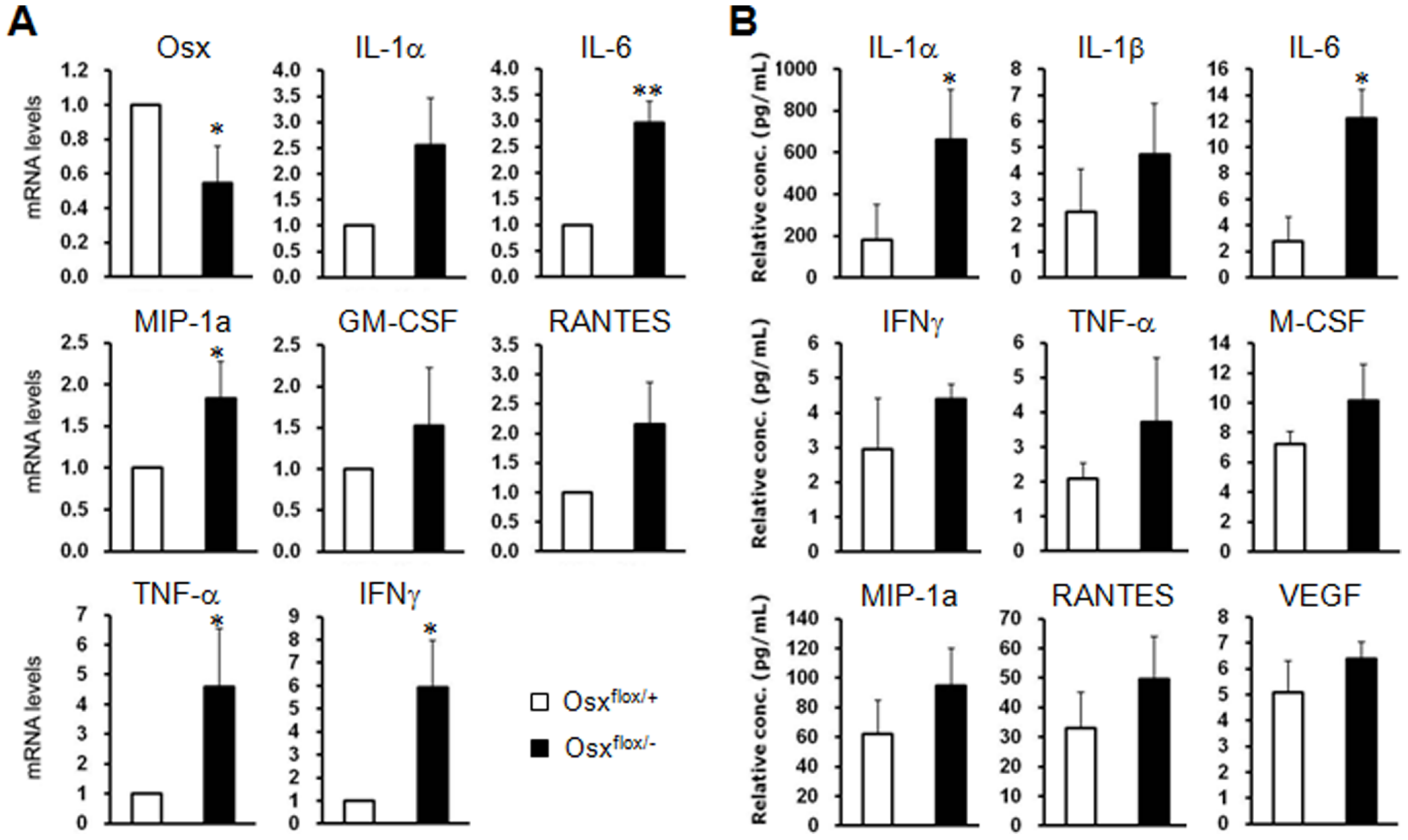
The inflammatory-prone condition induced by low Osx expression in Osx heterozygotes. (A) The mRNA expressions of the pro-inflammatory cytokines IL-6, TNF-α, and IFN-γ were elevated in bones of Osx heterozygous mice (Osx^flox/−^). *, p<0.05; **, p<0.01 versus wild-type. (B) Elevated serum levels of the proinflammatory cytokines, IL-1α and IL-6, were observed in Osx heterozygotes (Osx^flox/−^). *, p<0.05 versus wild-type.

Osteoblasts produce hormones and cytokines, and express genes that influence calcium and phosphate homeostasis and bone structure [6], [25], [26]. Therefore, we considered that a reduced ability of osteoblasts to form bone may explain the alterations of genes expressed and/or secreted in osteoblasts. To investigate whether the expressions of cytokines produced in osteoblasts were altered in Osx heterozygotes, the levels of various cytokines were assayed in bone and serum under normoxic conditions. In bone of Osx heterozygotes, the mRNA expressions of pro-inflammatory cytokines IL-6, TNF-α, and IFN-γ were significantly increased and those of others also exhibited increased expression (Fig. 2A and Fig. S2). In addition, the mRNA expression of the anti-inflammatory cytokine mouse IL-1 receptor antagonist (mIL-1ra) was decreased (data not shown). Furthermore, the serum levels of pro-inflammatory cytokines IL-1α and IL-6 were significantly increased in Osx heterozygotes (Fig. 2B). Those of the other pro-inflammatory cytokines IL-1β and TNF-α were increased in the serum of Osx heterozygotes to a slight, but statistically insignificant, amount. Even in an immunohistochemical analysis, IL-6, TNF-α, and IL-1α were highly expressed in bone tissue of Osx heterozygotes compared to wild-type (Fig. S3). Their expressions were relatively higher in conditional Osx knockout than Osx heterozygotes (Fig. S3), suggesting that the level of Osx expression may be important to regulate cytokine expression.

### Osx Down-regulates IL-6 Transcription in MC3T3-E1 Cells

**Figure 3:**
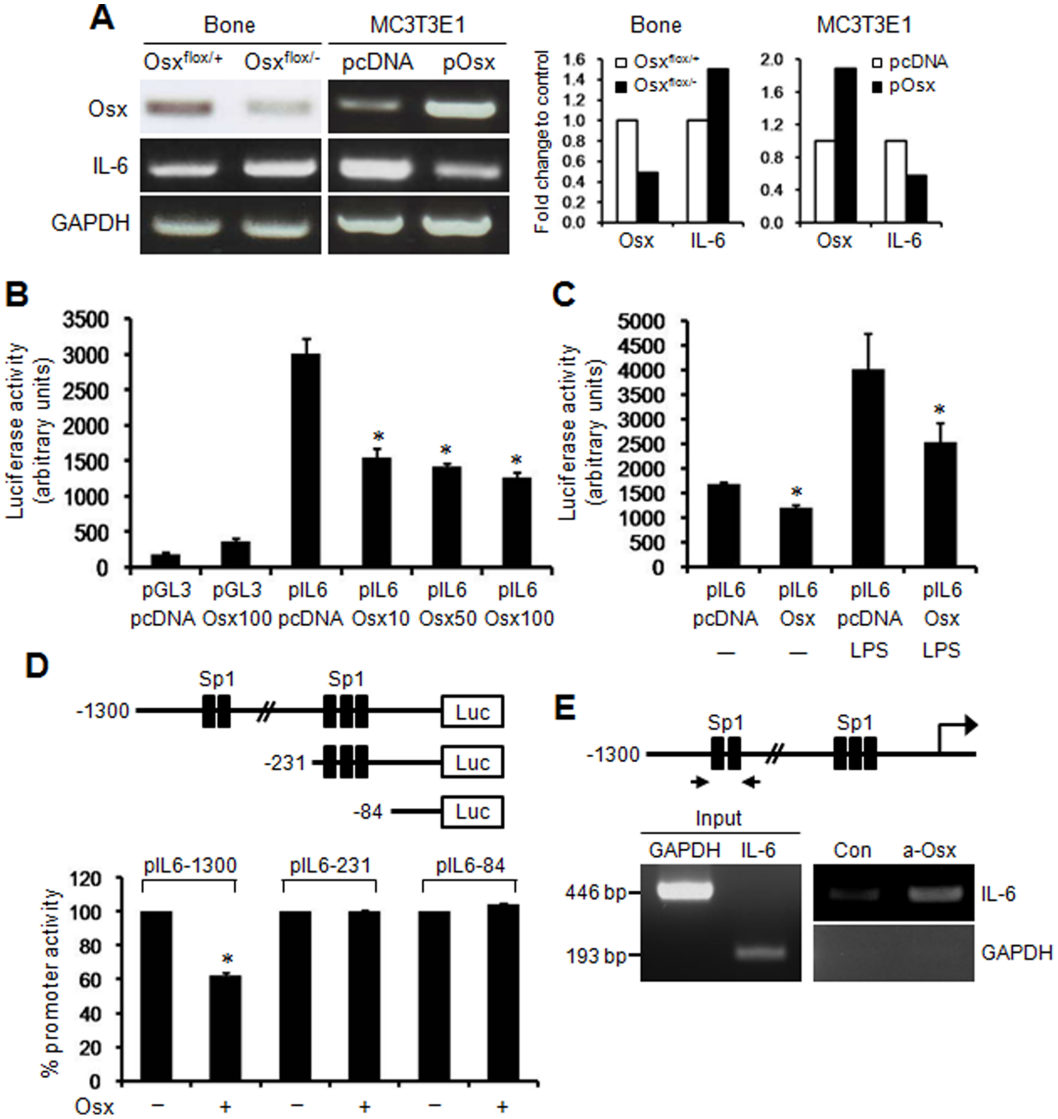
Negative regulation of IL-6 transcription by Osx in MC3T3-E1 cells. (A) The negative regulation of IL-6 by Osx expression. Compared to wild-type (Osx^flox/+^), Osx heterozygous mice (Osx^flox/−^) with low Osx expression exhibited increased IL-6 expression in bone at 7 wk of age. However, IL-6 levels were significantly lower in Osx overexpressing MC3T3-E1 cells. In the other hand, IL-6 expression was remarkably reduced in Osx overexpressing MC3T3E1 osteoblastic cells (pOsx) compared to control cells (pcDNA). The intensity of the individual bands of RT-PCR was determined using the Image J software. Data were normalized to GAPDH and expressed as fold change relative to control. (B) Osx significantly inhibited the transcriptional activity of IL-6 promoter in MC3T3-E1 osteoblastic cells. *, p<0.05 versus pcDNA control. (C) Osx inhibited LPS-induced IL-6 transcription in MC3T3-E1 cells. *, p<0.05 versus respective pcDNA control. (D) A schematic diagram of the full-length (-1300) and truncated (−231 and −84) IL-6 promoter-luciferase constructs. Osx inhibited the transcriptional activity of the full-length IL-6 promoter but not those of truncated IL-6 promoters. *, p<0.05. (E) Schematic representation of the Sp1 site in IL-6 promoter. Arrows indicate the primer set used for the ChIP assay. Chromatin prepared from MC3T3-E1 cells was immunoprecipitated with anti-Osx antibody (a-Osx) or isotype-matched control antibody (Con). The binding of Osx to the IL-6 promoter using immunoprecipitates was monitored by PCR using primers specific for the Sp1 site in IL-6 promoter. GAPDH was used as a negative control.

IL-6 is known to be a pro-inflammatory cytokine that is produced by osteoblasts which mediates chronic inflammation [10]. Osx expression was lower in the bone of Osx heterozygotes than in the wild-type, whereas IL-6 expression was significantly higher in the bone of Osx heterozygotes (Fig. 3A). To determine whether Osx regulates IL-6 expression, Osx was overexpressed in MC3T3-E1 osteoblastic cells. It was found that IL-6 expression was significantly lower in Osx overexpressing MC3T3-E1 cells than in pcDNA controls (Fig. 3A). These results indicated that reduced Osx expression in osteoblasts up-regulates a pro-inflammatory cytokine IL-6.

To investigate whether the expression of Osx inhibits IL-6 promoter activity, osteoblastic MC3T3-E1 cells were transiently transfected in the presence or absence of Osx. IL-6 transfected cells demonstrated a significant reduction of the luciferase activity in response to Osx, but not a dose-dependent reduction (Fig. 3B). We next examined whether Osx inhibits LPS-induced inflammation using a IL-6 promoter reporter. We found that the luciferase activity of the LPS-induced IL-6 promoter reporter was also inhibited by Osx (Fig. 3C). Osx, which belongs to the Sp/KLF superfamily, regulates the downstream target genes through Sp1 cognate elements as well as G/C-rich sequences [23], [27]. This IL-6 promoter contained two clusters of potential Sp1 sites for Osx binding existed. The first cluster contained two Sp1 sites, from −700 to −640 bp, and the second contained three Sp1 sites, from −130 to −90 bp. To identify Sp1 site required for Osx function, serially truncated (−231 and −84) IL-6 promoter-luciferase constructs were tested (Fig. 3D). Transcriptional activities of the truncated IL-6 promoters were not affected by adding Osx, indicating strongly that the first cluster of Sp1 containing binding sites may play a pivotal role in regulating IL-6 transcription by Osx. A promoter enzyme immunoassay and ChIP assay were carried out to confirm this result. In a promoter enzyme immunoassay, Osx bound significantly to the oligonucleotide containing the Sp1 binding site (Fig. S4). Mutations in Osx-responsive element attenuated the interaction between Osx and oligonucleotide probe. ChIP assay revealed that Osx directly bound to the first cluster of Sp1 sites of the IL-6 promoter (Fig. 3E). These results suggest that Osx suppresses IL-6 transcriptional activity through direct binding to the Sp1 sites of the IL-6 promoter.

### Reduced Kidney Repair in Osx Heterozygotes After I/R Injury

**Figure 4:**
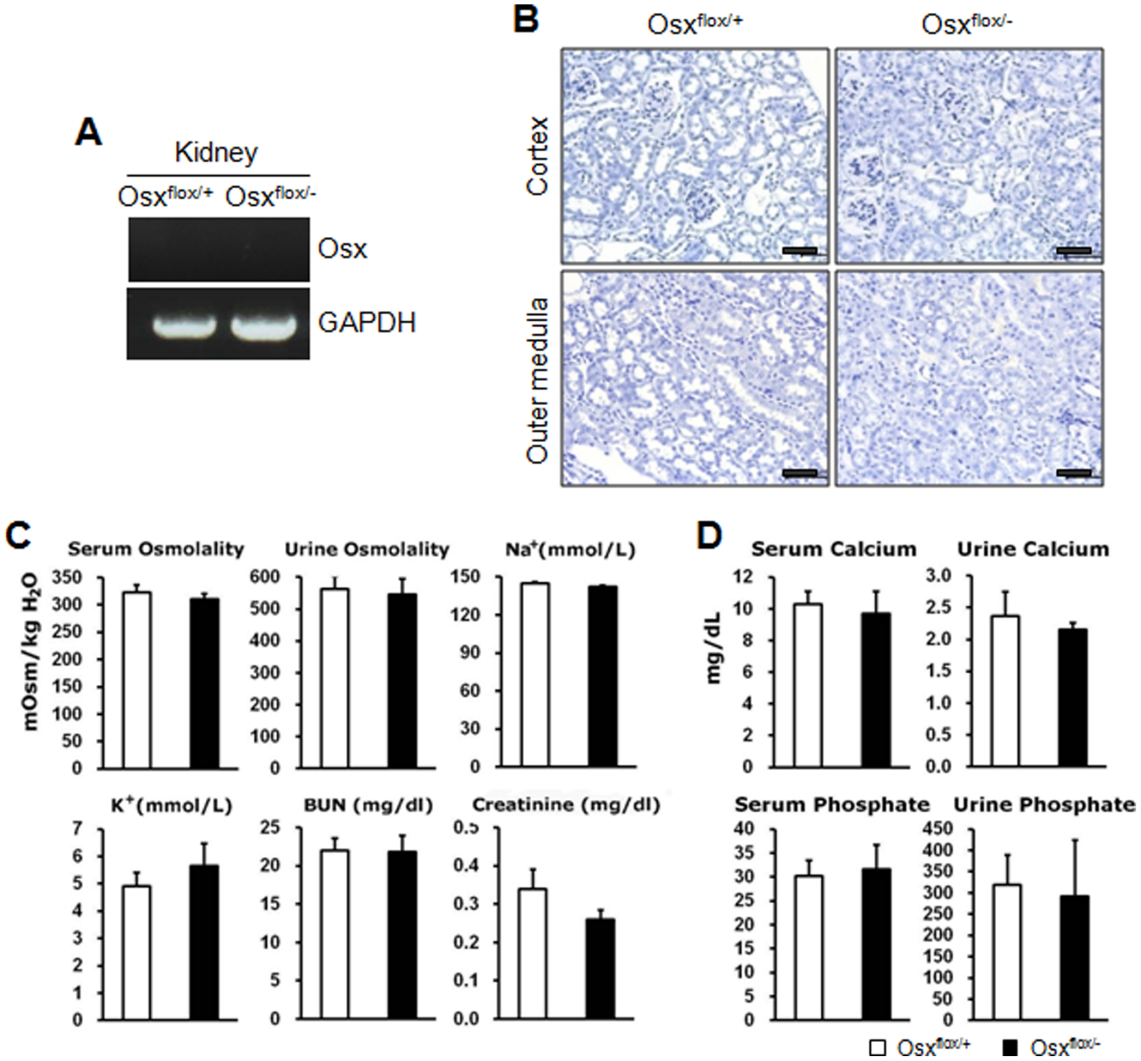
No alterations in renal function and morphology in Osx heterozygous mice. (A) The level of Osx expression in kidneys. Even in kidneys of wild-type (Osx^flox/+^), Osx expression was very low or negligible. (B) Morphologies of the renal cortex and outer medulla, stained with toluidine blue, were no different in Osx heterozygotes (Osx^flox/−^) and the wild-type. Scale bar = 50 µm. (C) No changes to indicators for renal function. Osmolality, Na^+^ and K^+^ concentrations, and blood urea nitrogen (BUN) and urine creatinine values were identical in both mouse populations. (D) Serum and urine calcium and phosphate levels were non-significantly different in both mouse populations.

The kidneys, like bone, importantly regulate the numerous endocrine factors necessary for maintaining a physiological balance of mineral ions in the body [28]. Furthermore, the kidneys are vulnerable to chronic inflammation [29], [30]. To determine the impact of reduced Osx expression on organ function, Osx expression was examined in the kidneys of Osx heterozygotes and the wild-type. Osx was primarily expressed in bone of the wild-type and, to a lesser degree, in the bone of Osx heterozygotes; Osx expression was negligible in the kidneys of wild-type and Osx heterozygous mice (Fig. 4A and Fig. S5A, B). Osx protein was also not detected in the kidneys of both mice (data not shown). No morphological alterations were observed in the renal cortex and outer medulla of Osx heterozygotes compared to wild-type (Fig. 4B). To evaluate renal function in Osx heterozygotes, several serum and urine parameters of Osx heterozygotes and wild-type were analyzed and compared. It was determined that there were no differences in serum and urinary osmolality, Na^+^ and K^+^ concentrations, BUN, and urine creatinine levels between Osx heterozygous and wild-type mice (Fig. 4C). Furthermore, the levels of calcium and phosphate were found to be similar in serum and urine samples from both mouse populations (Fig. 4D). Even though IL-6 was expressed, its expression exhibited an increased pattern without significant differences in kidneys of Osx heterozygotes compared to wild-type (Fig. S5A, B). Based on the increased IL-6 expression in the body, it has been questioned whether the JAK/STAT pathway is activated in kidney of Osx heterozygotes. No significant alterations were found in the JAK/STAT signals (Fig. S5C, D). These results indicated that Osx heterozygotes had no obvious renal functional defects under normoxic conditions.

**Figure 5:**
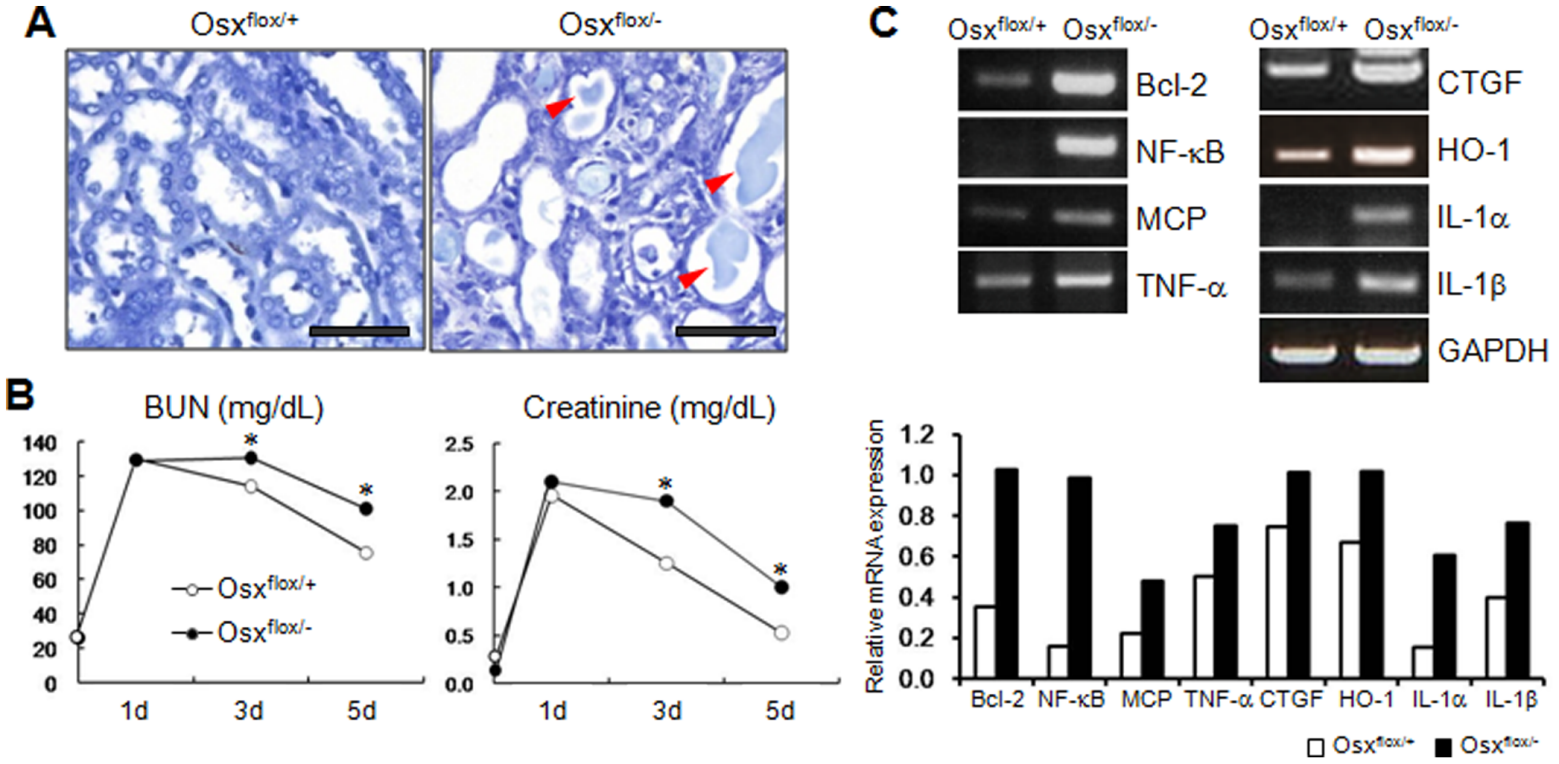
Reduced kidney repair function under diminished Osx expression. (A) Histological analysis of kidney subjected to ischemia/reperfusion (I/R) injury. Even after 5 days post I/R injury, the hypoxic kidneys from Osx heterozygotes (Osx^flox/−^) showed symptoms of acute tubular necrosis including the loss of epithelial brush border, epithelia flattening, and casts. Arrowheads indicates casts with necrotic and apoptotic tubular cells. Scale bar = 50 µm. (B) Delayed renal repair after I/R injury in Osx heterozygotes. BUN (an indicator of kidney failure and function) and creatinine levels remained higher after I/R injury in the kidneys of Osx heterozygotes than in those of the wild-type. *, p<0.05 versus wild-type (Osx^flox/+^). (C) Increased expressions of proinflammatory and fibrogenic cytokines in the hypoxic kidneys of Osx heterozygotes at 5 days after I/R injury. The expressions of proinflammatory and fibrogenic cytokines were elevated in the I/R injured kidneys of Osx heterozygotes. Relative mRNA expression levels of the genes were normalized to GAPDH and quantified using Image J software. Bcl2, B-cell lymphoma 2; NF-κB, nuclear factor kappa B; MCP, monocyte chemotactic protein; TNF-α, tumor necrosis factor alpha; CTGF, connective tissue growth factor; HO-1, Heme oxygenase 1; IL-1α, Interleukin-1 alpha; IL-1β, Interleukin-1 beta.

Chronic inflammation induced by high levels of pro-inflammatory cytokines has been reported to delay wound healing. Despite a normal renal function, Osx heterozygotes remained in an inflammatory-prone state, which makes them more susceptible to injury, and slow to repair damaged cells. To investigate the altered kidney repair mechanisms in the inflammatory-prone condition of Osx heterozygotes, acute renal failure induced by I/R injury was administered to wild-type and Osx heterozygous mice. At 5 days after I/R injury, renal morphologies were analyzed for both populations in kidney tissues stained with toluidine blue. In the wild-type, the tubular epithelial cells of the injured kidney were almost repaired at 5 days after I/R injury. However, it was found that the outer medullae of I/R administered kidneys were severely damaged in Osx heterozygotes, as indicated by acute tubular necrosis with the loss of the epithelial brush border, a flattened epithelium, and many casts with necrotic and apoptotic tubular cells (Fig. 5A). The relative kidney function was assessed by measuring BUN and creatinine levels on days 1, 3, and 5 after I/R injury (Fig. 5B). BUN values were less reduced in Osx heterozygotes than in the wild-type, indicating that repair of kidney was retarded in Osx heterozygotes after I/R injury. The concentrations of plasma creatinine exhibited the same pattern as BUN levels in I/R induced Osx heterozygotes. After I/R injury, however, the renal tissues of wild-type mice were restored to normal morphology and function (Fig. 5A, B). Furthermore, tubular cell proliferation (as determined by BrdU incorporation) was increased in Osx heterozygotes (data not shown), indicating that the damaged cells were more slowly regenerated, and thus, that renal repair was delayed. Numbers of TUNEL-positive apoptotic cells were not significantly different in Osx heterozygotes after I/R injury (data not shown). Inflammation-induced renal failure alters cytokine profiles and leads to the inflammatory cascade [31]. To assess the alteration of the cytokine profiles in I/R injured kidneys, we measured the expression levels of pro-inflammatory cytokine biomarkers. It was found that levels of pro-inflammatory and fibrogenic cytokine gene expressions were higher in the I/R injured kidneys of Osx heterozygotes (Fig. 5C), indicating the effects of persistent injuries in Osx heterozygotes. These results demonstrated that renal repair was delayed in Osx heterozygotes after I/R injury because of the inflammatory-prone state induced by low levels of Osx expression in bone.

## Discussion

The essential role of Osx in osteoblast differentiation and bone formation has been extensively documented. In a study conducted by Nakashima et al. [23], Osx homozygous null mutants died perinatally with no bone formation due to the complete arrest of osteoblast differentiation, suggesting that Osx is required for osteoblast differentiation during embryonic development. Baek et al. [22] found osteopenic bone architectures with delayed osteoblast maturation, accumulated immature osteoblasts, and reduced osteoblast function in terms of bone formation postnatally in osteoblast-specific Osx inactivated null mice, indicating that Osx is also necessary for adult bone formation. Likewise, the conditional ablation of Osx in osteoblasts via tamoxifen-induced Cre activity after birth resulted in a functional defect in osteoblasts and subsequent reduced bone formation, revealing the importance of Osx in postnatal bone formation and maintenance [32]. However, with the exceptions of osteoblast differentiation and bone formation, the functions of Osx have not been studied.

In the present study, Osx heterozygotes had normal bone shape but a weaken skeleton compared to the wild-type, as shown by peripheral QCT and in vitro osteoblast differentiation, indicating that the osteoblast function with respect to bone formation was reduced due to low levels of Osx expression in the bones of Osx heterozygotes. In addition to the expression of various bone transcription factors responsible for cell differentiation and bone formation [33], [34], osteoblasts also produce and secrete many cytokines [5], [6], [7], [8]. These cytokines, in turn, affect systemic inflammation [6], [8] and bone metabolism [7], [35], [36], which suggest regulatory linkages between bone transcription factors and cytokines. The pro-inflammatory cytokine TNF-α stimulates bone resorption and inhibits bone formation, and is also involved in systemic inflammation [37], [38], [39]. TNF-α-activated signaling is required for regulating the homeobox transcription factor Msx2 during osteoblast differentiation [40]. Furthermore, TNF-induced IL-6 expression in osteoblasts affects bone metabolism and turnover, and IL-6 release is suppressed by Wnt3a [41], [42]. Chronic inflammation caused by the high systemic levels of the pro-inflammatory cytokines, such as, IL-6 and IL-1β, increases IGFBP-3 expression and causes growth retardation [17]. The production of IL-1α, a pro-inflammatory cytokine with stimulatory effects on osteoclastogenesis, is suppressed by Osx in osteosarcoma cells. Thus, increased levels of IL-1α resulting from Osx inhibition gave rise to an altered tumor phenotype [21]. In the present study, IL-6 expression and secretion were increased in the bone and serum of Osx heterozygous mice, respectively, and its expression was significantly reduced in Osx overexpressing osteoblasts via a transcription-mediated mechanism, indicating that IL-6 production is suppressed by Osx expression in osteoblasts.

**Figure 6:**
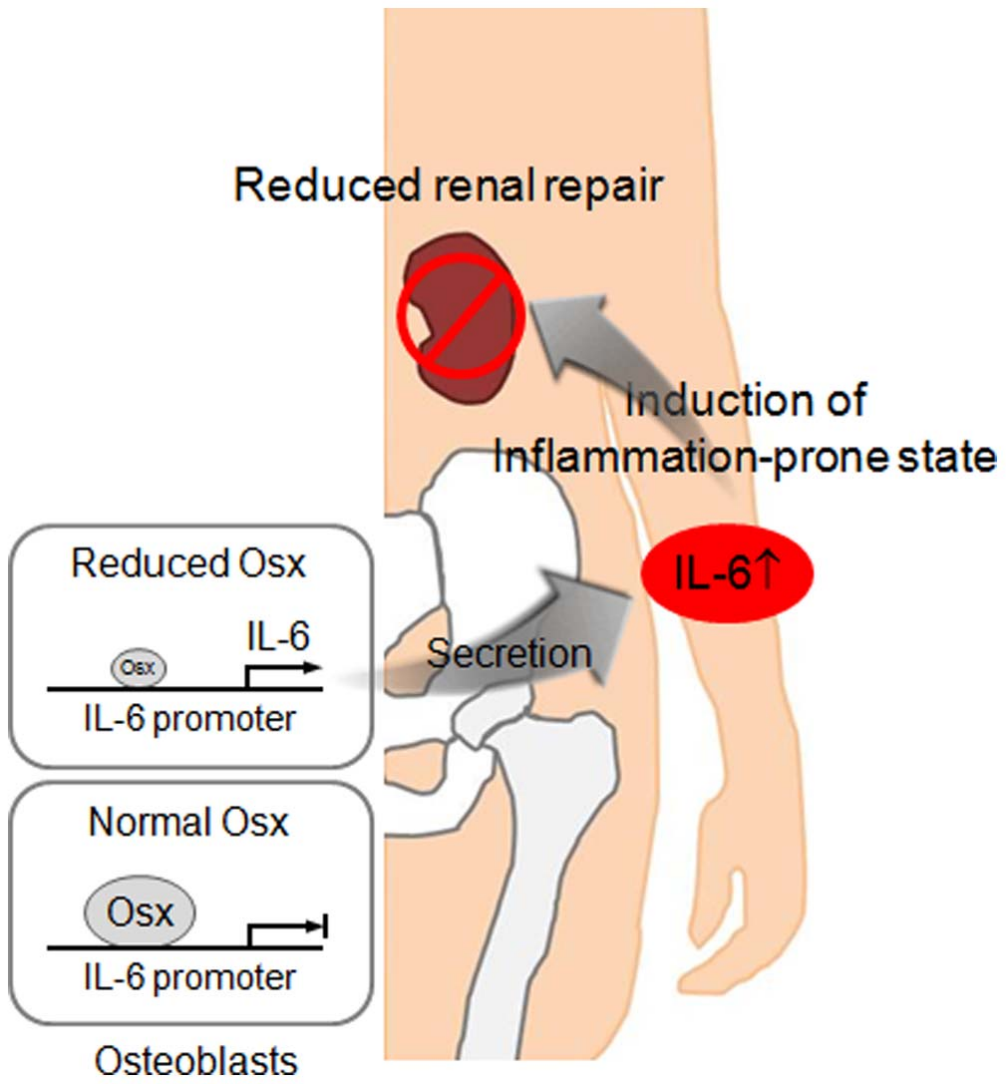
Proposed model of the role of Osx to prevent IL-6 production and an impairment of tissue regeneration. Persistent low level of Osx in bone accelerates the expression levels of various pro-inflammatory cytokines and leads to inflammatory-prone state of the body. In particular, reduced levels of Osx in osteoblasts fail to prevent IL-6 transcription, resulting in the accelerated production and secretion of IL-6, a key pro-inflammatory cytokine that mediates chronic inflammation. Finally, an inflammatory-prone state makes the kidneys more susceptible to injury, and delay to repair damaged cells. Thus, Osx, a negative regulator of IL-6 expression in osteoblasts, is important to prevent the establishment of an inflammatory-prone state and to regulate physiological homeostasis.

Pro-inflammatory cytokines have been found in parts of the immune systems that mediate direct biological functions and play important roles in the initiation and perpetuation of chronic inflammatory processes [8], [9]. High concentrations of pro-inflammatory cytokines have been reported to be related to the prognoses and developments of diseases. Moreover, chronic inflammation induced by elevated levels of pro-inflammatory cytokines delays wound healing. For example, the combination of IL-1 and TNF induces septic shock, resulting in a synergistic potentiation of sepsis [43]. The increased productions of IL-1β and IL-18 induce renal inflammation and diseases [44], and excessive IL-6 expression promotes the development and progression of chronic inflammatory diseases, such as, cardiovascular diseases, Alzheimer’s disease, rheumatoid arthritis, and CKD [11], [12], [13], [14]. Chronic inflammation induced by the upregulation of pro-inflammatory chemokines/cytokines accelerates ischemic brain injury [2]. A systemic allergy and asthma animal model with chronic inflammation displays an enhanced risk of acute myocardial I/R injury [1] and sickle mice with chronic inflammation show similar enhanced sensitivity to renal I/R injury [3]. Here, an inflammatory-prone state provoked by increased production of pro-inflammatory cytokines reduced renal repair in Osx heterozygous mice after I/R injury. As depicted in Fig. 6, these results indicate that an inflammatory-prone state is due to accelerated production and secretion of pro-inflammatory cytokines including IL-6 in Osx heterozygotes, resulting in enhanced susceptibility to renal injury and delayed renal repair. Taken together, this study provides the first experimental evidence supporting Osx as a modulator of inflammation, and thus provides novel insight of the potential use of Osx to prevent the systemic inflammatory-prone state.

## Supporting Information

Figure S1 **Expression patterns of osteoblast marker genes in bones of Osx heterozygotes by RT-PCR (A) and quantitative real-time PCR (B) analysis.** The intensity of the individual bands of RT-PCR was determined using the Image J software. Data were normalized to GAPDH and expressed as fold change relative to control. The expression of Osx was decreased by up to 50% in bone tissues of Osx heterozygotes (Osx^flox/–^) compared with wild-type (Osx^flox/+^) mice. In Osx^flox/–^ mice, the expressions of ALP and Col1 were obviously reduced by both analyses. ALP, alkaline phosphatase; Col1, type I collagen; Bsp, Bone sialoprotein; OCN, osteocalcin. *, p<0.05 versus Osx^flox/+^.
(TIF)

Figure S2 **Expression levels of pro-inflammatory and fibrogenc cytokines in bone of Osx heterozygotes by RT-PCR (A) and quantitative real-time PCR (B) analysis.** The intensity of the individual bands of RT-PCR was determined using the Image J software. Data were normalized to GAPDH and expressed as fold change relative to control. The mRNA expressions of the examined cytokines were increased in bones of Osx heterozygotes (Osx^flox/−^) compared with wild-type (Osx^flox/+^) mice. *, p<0.05 versus Osx^flox/+^.
(TIF)

Figure S3 **Immunohistochemical analysis of Osx and pro-inflammatory cytokines expression in bone.** (A) Osx expression was not observed in bone tissue of conditional Osx knockout (Osx^flox/–^;Col1a1-Cre) compared to Osx heterozygotes (Osx^flox/–^). (B) IL-6, TNF-α, and IL-1α were expressed in bone tissue including osteoblasts, osteocytes, and chondrocytes. Their expressions were increased in Osx^flox/–^ and more increased in Osx^flox/–^;Col1a1-Cre than wild-type (Osx^flox/+^). Scale bar = 50 μm.
(TIF)

Figure S4 **Promoter enzyme immunoassay.** Oligonucleotide probes correspond to a wild-type or mutated Osx-responsive element. Mutations in Osx-responsive element are underlined. Wild-type and mutated oligonucleotides were conjugated onto streptavidin-coated 96-well plates, and nuclear extracts from 293FT cells transfected with the Osx expression vector were added. After incubation for 2 h, interaction between Osx and probe was analyzed using anti-Osx antibody and HRP-conjugated secondary antibody. Result represents the mean ± S.D. of three independent experiments. t-test: *, p<0.05.
(TIF)

Figure S5 **Expression patterns of Osx, IL-6, and genes related to JAK/STAT signaling in kidneys.** (A, B) Osx and IL-6 expression in kidneys of Osx heterozygotes (Osx^flox/–^) by RT-PCR (A) and quantitative real-time PCR (B) analysis. Osx was not expressed in the kidneys of both mice. IL-6 expression exhibited an increased pattern with no significance in the kidneys of Osx^flox/–^ compared to wild-type (Osx^flox/+^). (C, D) Expression patterns of genes related to the JAK/STAT signaling in kidneys of Osx^flox/–^ by RT-PCR (C) and quantitative real-time PCR (D) analysis. The intensity of the individual bands of RT-PCR was determined using the Image J software. Data were normalized to GAPDH and expressed as fold change relative to control. While ERK1 expression revealed an increased pattern, the expressions of gp130 and AKT1 genes showed a reduced pattern in kidneys of Osx^flox/–^ by the analysis of quantitative real-time PCR. However, no significant alterations of expression levels were examined.
(TIF)

Text S1 **Supporting Materials and Methods.**
(DOC)
