# Supplementary figures and images for: Persistent Low Level of Osterix Accelerates Interleukin-6 Production and Impairs Regeneration after Tissue Injury
Source: PLoS One. 2013 Jul 26;8(7):e69859. doi: 10.1371/journal.pone.0069859 (PMC3724732; doi:10.1371/journal.pone.0069859)

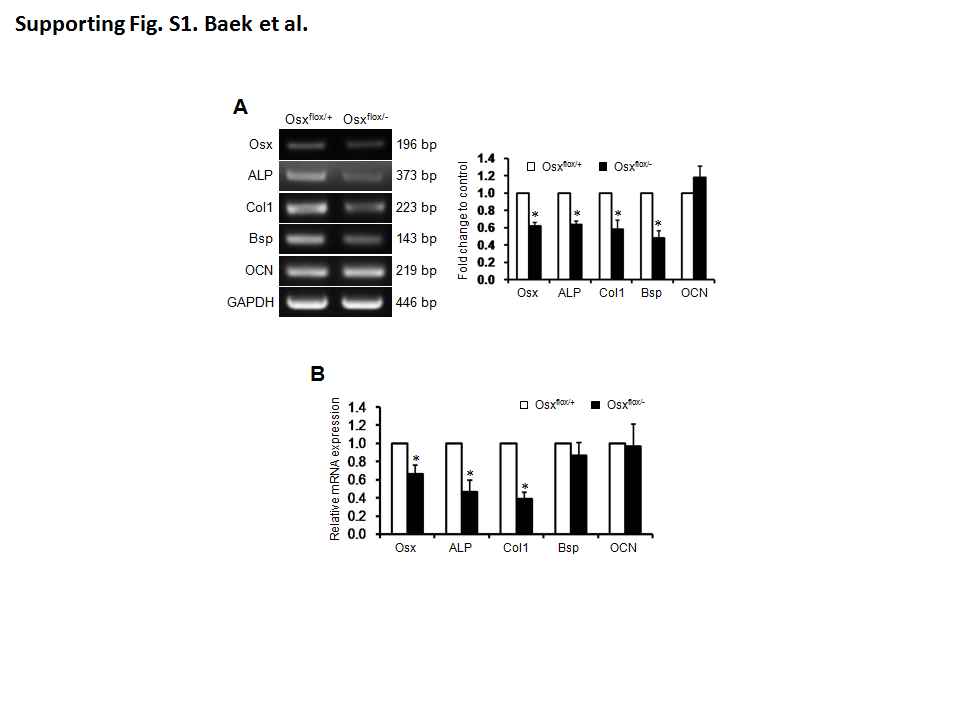

Supplement: Figure S1 — Expression patterns of osteoblast marker genes in bones of Osx heterozygotes by RT-PCR (A) and quantitative real-time PCR (B) analysis. The intensity of the individual bands of RT-PCR was determined using the Image J software. Data were normalized to GAPDH and expressed as fold change relative to control. The expression of Osx was decreased by up to 50% in bone tissues of Osx heterozygotes (Osxflox/–) compared with wild-type (Osxflox/+) mice. In Osxflox/– mice, the expressions of ALP and Col1 were obviously reduced by both analyses. ALP, alkaline phosphatase; Col1, type I collagen; Bsp, Bone sialoprotein; OCN, osteocalcin. *, p<0.05 versus Osxflox/+. (TIF) [file pone.0069859.s001.tif]

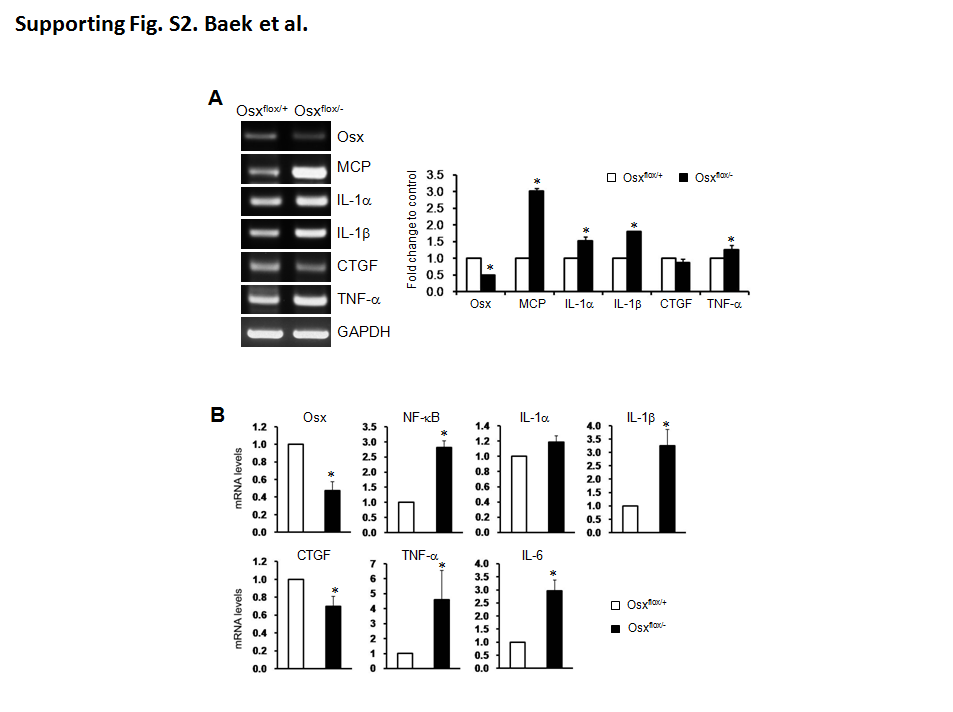

Supplement: Figure S2 — Expression levels of pro-inflammatory and fibrogenc cytokines in bone of Osx heterozygotes by RT-PCR (A) and quantitative real-time PCR (B) analysis. The intensity of the individual bands of RT-PCR was determined using the Image J software. Data were normalized to GAPDH and expressed as fold change relative to control. The mRNA expressions of the examined cytokines were increased in bones of Osx heterozygotes (Osxflox/−) compared with wild-type (Osxflox/+) mice. *, p<0.05 versus Osxflox/+. (TIF) [file pone.0069859.s002.tif]

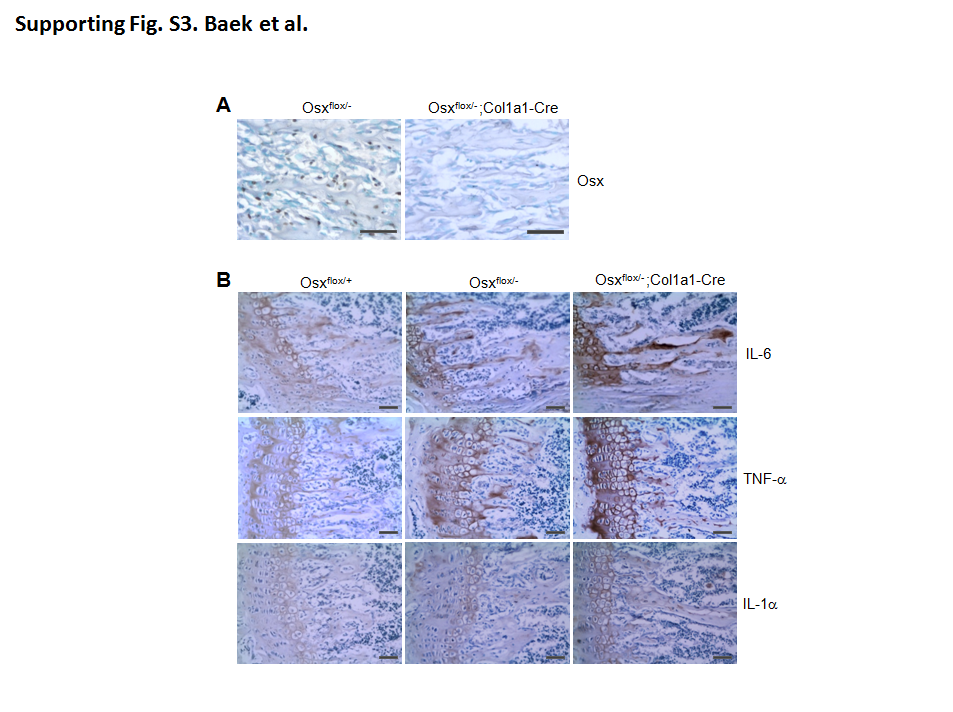

Supplement: Figure S3 — Immunohistochemical analysis of Osx and pro-inflammatory cytokines expression in bone. (A) Osx expression was not observed in bone tissue of conditional Osx knockout (Osxflox/–;Col1a1-Cre) compared to Osx heterozygotes (Osxflox/–). (B) IL-6, TNF-α, and IL-1α were expressed in bone tissue including osteoblasts, osteocytes, and chondrocytes. Their expressions were increased in Osxflox/– and more increased in Osxflox/–;Col1a1-Cre than wild-type (Osxflox/+). Scale bar = 50 μm. (TIF) [file pone.0069859.s003.tif]

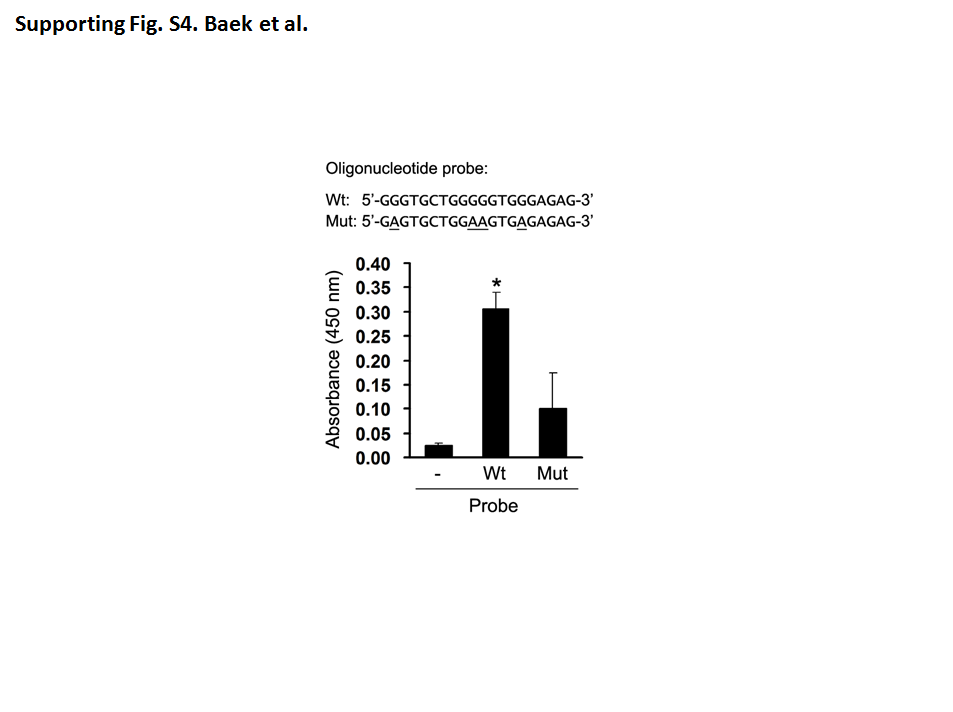

Supplement: Figure S4 — Promoter enzyme immunoassay. Oligonucleotide probes correspond to a wild-type or mutated Osx-responsive element. Mutations in Osx-responsive element are underlined. Wild-type and mutated oligonucleotides were conjugated onto streptavidin-coated 96-well plates, and nuclear extracts from 293FT cells transfected with the Osx expression vector were added. After incubation for 2 h, interaction between Osx and probe was analyzed using anti-Osx antibody and HRP-conjugated secondary antibody. Result represents the mean ± S.D. of three independent experiments. t-test: *, p<0.05. (TIF) [file pone.0069859.s004.tif]

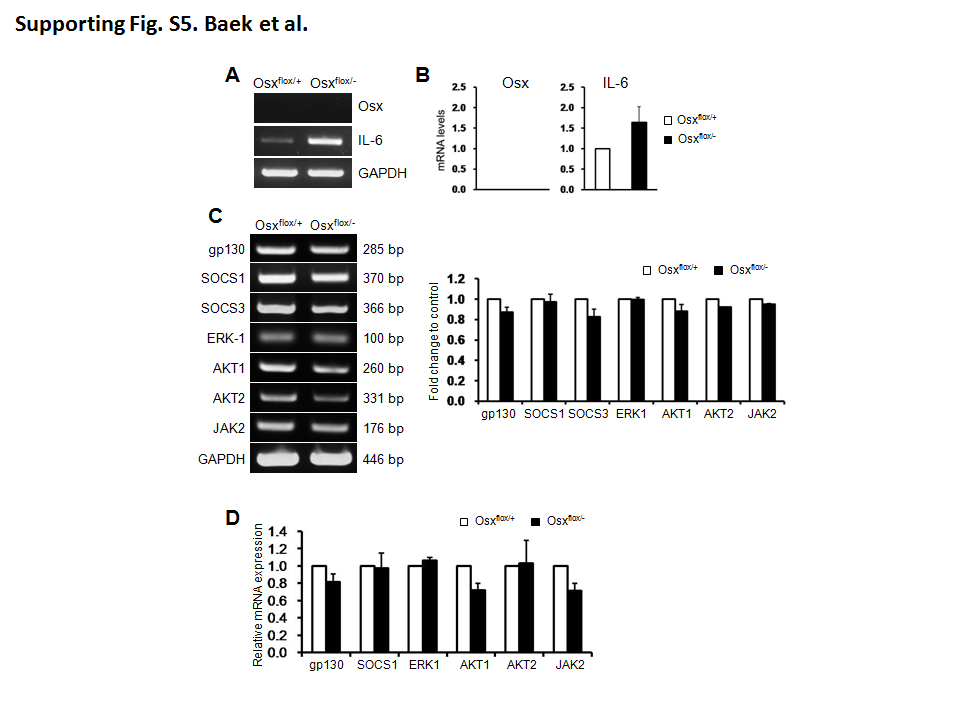

Supplement: Figure S5 — Expression patterns of Osx, IL-6, and genes related to JAK/STAT signaling in kidneys. (A, B) Osx and IL-6 expression in kidneys of Osx heterozygotes (Osxflox/–) by RT-PCR (A) and quantitative real-time PCR (B) analysis. Osx was not expressed in the kidneys of both mice. IL-6 expression exhibited an increased pattern with no significance in the kidneys of Osxflox/– compared to wild-type (Osxflox/+). (C, D) Expression patterns of genes related to the JAK/STAT signaling in kidneys of Osxflox/– by RT-PCR (C) and quantitative real-time PCR (D) analysis. The intensity of the individual bands of RT-PCR was determined using the Image J software. Data were normalized to GAPDH and expressed as fold change relative to control. While ERK1 expression revealed an increased pattern, the expressions of gp130 and AKT1 genes showed a reduced pattern in kidneys of Osxflox/– by the analysis of quantitative real-time PCR. However, no significant alterations of expression levels were examined. (TIF) [file pone.0069859.s005.tif]
