# Supplementary material for: Persistent Low Level of Osterix Accelerates Interleukin-6 Production and Impairs Regeneration after Tissue Injury
Source: PLoS One. 2013 Jul 26;8(7):e69859. doi: 10.1371/journal.pone.0069859 (PMC3724732; doi:10.1371/journal.pone.0069859)
Supplement: Text S1 — Supporting Materials and Methods. (DOC) [file pone.0069859.s006.doc]

**Text S1. Supporting Materials and Methods**

RT-PCR and quantitative real-time PCR

Total RNA was isolated from long bones and kidneys using Tri reagent (Invitrogen, Camarillo, CA, USA) and used to synthesize cDNA using Reverse Transcriptase Premix (Elpis-Biotech, Daejeon, Korea) according to the manufacturer’s instructions. The primers used for RT-PCR and quantitative real-time PCR were as follows: alkaline phosphatase (ALP), 5'-GCC CTC TCC AAG ACA TAT A-3' and 5'-CCA TGA TCA CGT CGA TAT CC-3'; type I collagen (Col1), 5'-CCT GGT AAA GAT GGT GCC-3' and 5'-CAC CAG GTT CAC CTT TCG CA-3'; Bone sialoprotein (Bsp), 5'-AAG CAG CAC CGT TGA GTA TGG-3' and 5'-CCT TGT AGT AGC TGT ATT CGT CCT C-3'; osteocalcin (OCN), 5'-CCT CAG TCC CCA GCC CAG ATC C-3' and 5'-CAG GGC AGA GAG AGA GGA CAG G-3'; gp130, 5'-TCG GAG GAG CGG CCA GAA GAC-3' and 5'-ATC AGC CCC CGT GCC AAG AGC-3'; SOCS1, 5'-CTC GAG TAG GAT GGT AGC ACG CAA-3' and 5'-CAT CTT CAC GCT GAG CGC GAA GAA-3'; ERK-1, 5'-CTG GAC CAG CTC AAC CAC ATT-3' and 5'-AGA GAC TGC AGG TAG TTT CGG G-3'; AKT1, 5'-GCC TAC CGA GAA GAG ACT CTG A-3' and 5'-GTC TTC ATC AGC TGG CAT TGT-3'; AKT2, 5'-TAA AAA GTG GCT CTG GTG TGT G-3' and 5'-GGC ATT CTG CTA CAG AGA AAT TG-3'; and JAK2, 5'-GAT GGC GGT GTT AGA CAT GA-3' and 5'-TGC TGA ATG AAT CTG CGA AA-3'.

Immunohistochemistry

For the immunohistochemical analysis, wild-type (Osx^flox/+^), Osx heterozygotes (Osx^flox/–^), and osteoblast-specific conditional Osx knockout (Osx^flox/–^;Col1a1-Cre) [1] were used. Immunostaining was performed with Histostain Plus Kit (Invitrogen, Camarillo, CA, USA) following the manufacturer's instruction. Briefly, 5-μm sections from 4% PFA-fixed paraffin-embedded blocks were deparafinized in xylene and rehydrated through graded ethanol series. Endogenous peroxidase activity was quenched in 0.3% H_2_O_2_ in methanol. After blocking at room temperature for 1 h, the sections were incubated with primary antibodies to Osx (ab22552, Abcam, Cambridge, UK; dilution 1:100), IL-6 (ab6672; dilution 1:250), TNF-α (ab6671; dilution 1:100), and IL-1α (ab7632; dilution 1:50) in 1× phosphate-buffered saline (PBS) for 1 h at 4°C. Signals for antibody binding were visualized with diaminobenzidine (DAB) substrate (Invitrogen) and counterstaining was performed with 0.25% methyl green.

Promoter enzyme immunoassay

For biotin-labeled probes, sense and antisense oligonucleotides containing wild-type or mutant Osx-responsive elements were synthesized, combined in an equimolar mixture, and annealed for 1 h at 37°C to form the double-stranded probe. The sequences of the oligonucleotides used were as follows: sense probe, 5'-Biotin-GGG TGC TGG GGG TGG GAG AG'; antisense probe, 5'-CTC TCC CAC CCC CAG CAC CC-3'; sense mutant probe, 5'-Biotin-GAG TGC TGG AAG TGA GAG AG-3'; antisense mutant probe, 5'-CTC TCT CAC TTC CAG CAC TC-3'. Promoter enzyme immunoassays were performed as previously described [2] with slight modifications. Streptavidin-coated 96-well plates (Thermo Fisher Scientific) were washed three times with PBS-T buffer (PBS and 0.05% Tween 20) and then incubated with biotin-labeled probes containing wild-type or mutant Osx-responsive elements (5 pmol/well) for 1 h at room temperature. After three washes with PBS-T buffer, the plate was incubated with the nuclear extract (10 μg/well) from the 293FT cells transfected with the Osx expression vector and poly-deoxyinosinic-deoxycytidylic acid (10 μg/well) at 4°C for 2 h. The plates were washed with HKMG buffer [10 mM HEPES (pH 7.9), 100 mM KCl, 5 mM MgCl_2_, 10% glycerol, 1 mM DTT, 0.5% Nonidet P-40], and incubated with anti-Osx antibody (Abcam) at 4°C for 2 h. After three washes with HKMG buffer, HRP-conjugated anti-rabbit IgG was added, and the plates were then incubated for 1 h at 4°C. The colorimetric reaction was developed by adding a substrate reagent (R&D Systems) and quantified by measuring the absorbance at 450 nm.

**Supporting Reference**

1. Baek WY, Lee MA, Jung JW, Kim SY, Akiyama H, de Crombrugghe B, Kim JE (2009) Positive regulation of adult bone formation by osteoblast-specific transcription factor osterix. J Bone Miner Res 24: 1055-1065.
2. Klunker S, Chong MM, Mantel PY, Palomares O, Bassin C, Ziegler M, Ruckert B, Meiler F, Akdis M, Littman DR, Akdis CA (2009) Transcription factors RUNX1 and RUNX3 in the induction and suppressive function of Foxp3+ inducible regulatory T cells. J Exp Med 206: 2701-2715.
